# The Influence of Intrinsic Motivation on Pay Satisfaction Among Caregivers in Residential Home for the Elderly in China: The Mediating Role of Job Burnout

**DOI:** 10.1097/jnr.0000000000000399

**Published:** 2020-08-14

**Authors:** Enjian WANG, Hongwei HU, Hongting LIU, Shan MAO, Ruihua CHANG, Wei JIANG

**Affiliations:** 1 PhD, Associate Professor, School of Humanities and Social Sciences, North China Electric Power University, Baoding, Hebei Province, PRC; 2 PhD, Associate Professor, School of Public Administration and Policy, Renmin University of China, Beijing, PRC; 3 BS, School of Public Administration and Policy, Renmin University of China, Beijing, PRC; 4 BS, School of Sociology and Population Studies, Renmin University of China, Beijing, PRC; 5 Undergraduate, School of Humanities and Social Sciences, North China Electric Power University, Baoding, Hebei Province, PRC.

**Keywords:** caregiver, intrinsic motivation, job burnout, pay satisfaction, China

## Abstract

**Background:**

According to the theory of compensating differentials, caregivers with high levels of intrinsic motivation should exhibit a higher-than-average satisfaction with their pay. Whereas studies conducted in Western countries have provided empirical evidence for the theory of compensating differentials in various care settings, few studies have been conducted in China that focus on caregivers employed in residential homes for the elderly (RHE). The sociodemographic characteristics of caregivers in China different significantly from their counterparts in Western countries.

**Purpose:**

This study was developed to analyze the mediating role of job burnout to assess the influence of intrinsic motivation on pay satisfaction among caregivers in RHE.

**Methods:**

Structural equation modeling was used to examine the influences of intrinsic motivation on pay satisfaction in a sample of 1,212 caregivers employed in RHE in China by analyzing the mediating role of job burnout.

**Results:**

Intrinsic motivation was found to relate positively to pay satisfaction (β = .11, *p* < .05). Negative relationships were identified between intrinsic motivation and job burnout (β = −.46, *p* < .01) and between job burnout and pay satisfaction (β = −.13, *p* < .01). Job burnout was found to have a significant mediating effect on the relationship between intrinsic motivation and pay satisfaction (β = .06, *p* < .01).

**Conclusions/Implications for Practice:**

A significant relationship was found between intrinsic motivation and pay satisfaction, with job burnout playing a mediating role in caregivers employed in RHE in China. This research has profound implications for nursing education, practice, and research. First, greater efforts should be focused on instilling nursing values in nursing students to foster intrinsic motivation. Second, nonpecuniary rewards may be offered to caregivers to acknowledge the values of care work and strengthen intrinsic motivation. Third, a supportive working climate should be fostered to reduce job burnout. Fourth, caregivers should be informed of their rights to decent pay and their right to bargain collectively through unions. Fifth, appropriate public policies should be implemented to provide pay for caregivers at levels that recognize and appreciate their intrinsic motivation.

## Introduction

The rapidly aging population in China has significantly increased need for elderly caregivers in residential homes for the elderly (RHE). However, critical shortages of these caregivers coupled, and a turnover rate for caregivers working in RHE have been widely reported (Fu, 2018). These problems are attributed in large part to the wage penalty, meaning that caregivers are generally paid less than other occupations; net of human capital differences; and demographic characteristics (England et al., 2002). The wage penalty of caregivers has been attributed to a variety of issues, including the cultural devaluation of care work because of its association with “women's work” (Magnusson, 2008), the economic dependence of care recipients who need care services the most but are the least able to pay for these services (England et al., 2002), the limited bargaining power that caregivers have to demand higher wages (Dong et al., 2017), the lower requirements of care work in terms of human capital and skill (Budig & Misra, 2011), and the difficulty in achieving productivity gains in the care sector (England et al., 2002).

However, these explanations generally ignore the perceptions of caregivers regarding their low pay status. According to the theory of compensating differentials, if caregivers consider the intrinsic nature of caring work as an amenity for themselves, they may self-justify their wage penalty, be more satisfied with their wages, and be willing to accept lower wages (England, 2005; England et al., 2002). In other words, the nonpecuniary benefits of caring work may partially compensate for low pay, which may result in a disinclination of caregivers to bargain for higher pay and give employers an incentive to maintain low wages. Although some studies have provided strong empirical evidence for the theory of compensating differentials in the care labor market with respect to caregivers in various settings (Fryer, 2013; Nelson & Folbre, 2006), few have been conducted that focus on caregivers employed in RHE, who are generally less paid and more likely to be perceived as unskilled, nonprofessional, and kindly “angels” (Nelson & Folbre, 2006). Moreover, previous studies were based on data mainly from Western countries. On the basis of a search by the authors, no study in China has addressed this subject, raising questions regarding the validity in China of the findings of previous studies. The employment situation for caregivers in RHE in China differs significantly from that in Western countries. Most elderly caregivers employed in RHE in China are old, poorly educated, and from rural areas. Many are not certified and, therefore, are easily replaced. Moreover, because of the cultural influence of filial piety, elderly caregivers in RHE in China are more likely to be intrinsically motivated. The quality of affective relationships between elderly nurses and their elderly patients is often more valued by RHE managers than nursing skills as a guarantee of quality of care. Thus, the unique sociodemographic characteristics of geriatric nurses employed in RHE may impact the validity in China of predictors of turnover intention developed based on the conditions in Western countries.

In addition, in consideration of the influences of intrinsic motivation in care work on quality of care and turnover intention and that caregivers are very much underpaid by RHE, it is important to investigate the relationship between intrinsic motivation and pay satisfaction among caregivers employed in RHE in China. Therefore, this article was developed to investigate the influence of intrinsic motivation on pay satisfaction among caregivers employed in RHE. Furthermore, considering the significant relationships between intrinsic motivation and job burnout and between job burnout and pay satisfaction (Zarei Matin et al., 2012), this study further analyzed the mediating role of job burnout.

### Literature Review

#### Intrinsic motivation and pay satisfaction

Workers may be affected by intrinsic and extrinsic motivations. Intrinsic motivation may play a more important role in care activities as care work involves more nonpecuniary amenities than many other forms of work (Folbre & Smith, 2017). In the context of care work, intrinsic motivation encourages workers to engage in caregiving for personal reasons/priorities based on a sense of moral obligation, meaningfulness and fulfillment, autonomy at work, and/or affective relations with care recipients (England, 2005) and conformity to caring norms by caregiving itself (Folbre & Smith, 2017) rather than for extrinsic rewards. This form of motivation reflects a desire to engage in care work because an individual finds the work pleasant, is motivated by feelings of love/attachment, and/or feels this work is socially valuable (Folbre & Nelson, 2000). Self-determination theory distinguishes intrinsic motivation into the three types of autonomy, relatedness, and competence (Deci & Ryan, 2016) and typically measures intrinsic motivation of a workforce using autonomy (e.g., “Because it is the type of work I have chosen to attain certain important objectives.”; Tremblay et al., 2009), enjoyment (e.g., “Because I enjoy it.”; Grant et al., 2011), interest (e.g., “Because it's fun.”; Grant et al., 2011), fulfillment (e.g., “Because this job fulfills my career plans.”; Gagné et al., 2010), and consistency (e.g., “Because my work is a big part of who I am.”; Moran et al., 2012). According to the theory of compensating differentials, the intrinsic (nonmonetary) rewards gained from performing work may offset the expected dissatisfaction with low pay. Thus, caregivers who are motivated mainly by intrinsic motivation should be willing to sacrifice financial rewards for intrinsic rewards and be more satisfied with their pay in the caring labor market. Nelson and Taylor found that nurses with high levels of intrinsic motivation were more satisfied with their pay (Nelson & Folbre, 2006). Drawing on a case study conducted in a private residential care home, Johnson (2015) found that care workers justified the economic devaluation of their work through altruistic motivation. Hence, the following hypothesis is proposed in this study:

Hypothesis 1: Intrinsic motivation is positively related to pay satisfaction in caregivers employed in RHE.

#### Intrinsic motivation and job burnout

The generally accepted definition of job burnout is “a psychological syndrome emerging as a prolonged response to chronic interpersonal stressors on the job.” Job burnout includes the following three dimensions: (a) emotional exhaustion, interpreted as emotional depletion and loss of energy; (b) depersonalization, described as dehumanization, detachment from work and clients, and emotional hardening; and (c) reduced personal accomplishment or inefficacy, that is, a feeling of personal or professional inadequacy and reduced productivity and coping skills (Salvagioni et al., 2017). The findings of many studies indicate that intrinsic work motivation relates negatively to job burnout (Kuvaas et al., 2017). Intrinsically motivated workers are more self-determined (Beek et al., 2011), have more positive psychological adjustments (Brummelhuis et al., 2011), have better physical well-being (Fernet et al., 2013), exhibit more autonomy and competence (Ryan & Deci, 2000), cope with stress more effectively (Gagné et al., 1997), and are better able to balance their work and family lives (Senécal et al., 2001), all of which may buffer, reduce, or even eliminate job burnout. Hence, the following hypothesis is proposed:

Hypothesis 2: Intrinsic motivation is negatively related to job burnout in caregivers employed in RHE.

#### Job burnout and pay satisfaction

Job burnout in workers negatively impacts their well-being. A close relationship has been found between job burnout and continual stress, overwhelming psychological exhaustion, fatigue, depression, low self-esteem, health problems, sleep problems, and reduced tolerance to frustration (Adriaenssens et al., 2015), which implies that workers experiencing high levels of job burnout work in poor/undesirable conditions. As posited in the theory of compensating differentials, nonpecuniary benefits compensate somewhat for low pay and promote satisfaction with pay. By contrast, reducing nonpecuniary benefits should be offset by an increase in monetary compensation. As a result, the negative effect of job burnout should reduce the satisfaction of workers with their current pay. Hence, the following hypotheses are proposed:

Hypothesis 3: Job burnout is negatively related to pay satisfaction in caregivers employed in RHE.
Hypothesis 4: Intrinsic motivation has an indirect and negative impact on pay satisfaction that is mediated by job burnout in caregivers employed in RHE.

**Figure 1:**
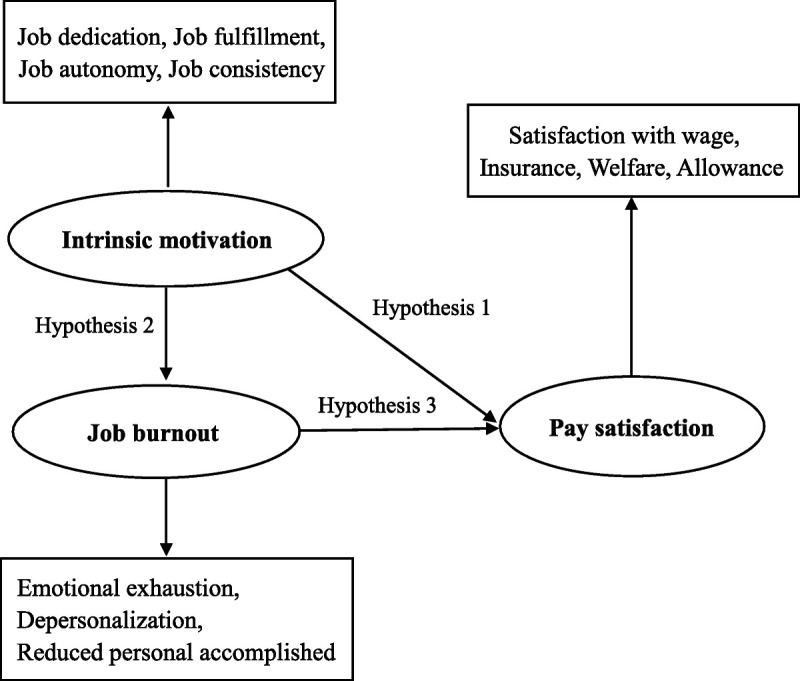
Proposed Theoretical Model

On the basis of the aforementioned hypotheses, the hypothetical model shown in Figure 1 was developed.

## Methods

### Setting and Sample

Caregivers employed in RHE in China are individuals who care for older adults in RHE who require supervision or assistance and are required to have professional ethics and a certain level of basic knowledge (Ministry of Civil Affairs of the People's Republic of China, 2007). Certification is not required for performing caregiving work in RHE. Caregivers may provide four kinds of care, including daily care, technical care, rehabilitation care, and psychological care. However, most caregivers provide only daily care and some technical care in practice. A multistage stratified random sampling approach using a typical area sampling design was adopted in this study. After assessing levels of social and economic development, 27 cities in 22 provinces were chosen from western, central, and eastern China. Information on the types (public and private) and sizes (“small nursing homes” with less than 50 beds, “medium nursing homes” with 51–199 beds, “large nursing homes” with 200–299 beds, and “extra-large nursing homes” with more than 300 beds) of nursing homes in each of the selected cities was gathered, and then six to eight nursing homes were selected. In each of the selected nursing homes, a questionnaire and an accompanying introductory letter that explained the purpose of the study and privacy protection for the participants were administered to caregivers after ethics approval was granted (on June 1, 2018). The questionnaire was designed by experienced nursing experts following a strict procedure to ensure the validity and reliability of the measurements. Mature scales with high validity and reliability that have been confirmed in many studies in China were used in this questionnaire. A pilot study was conducted with 30 caregivers from four types of RHE. On the basis of the results of the pilot study, the wordings of 18 questions were modified. Considering the limited educational levels of the participants, one-on-one interviews using the questionnaire were conducted. Well-trained investigators read each item to the participant without any explanation and then recorded her or his answer. If the participant did not hear the item clearly or understand what the item meant, the investigator repeated the item once. If the participant still did not understand what the item meant, the investigator provided minimal additional explanation without guidance regarding how that item should be answered. One thousand two hundred twelve geriatric nurses participated in this study. Data collection started in May 2018 and ended in June 2018.

### Measurements

#### Intrinsic motivation

Intrinsic motivation included four variables: job dedication, job fulfillment, job autonomy, and job consistency. Job dedication was assessed using a three-item subscale from the Utrecht Work Engagement Scale (Schaufeli & Bakker, 2004; α = .833), including the statements “I am full of enthusiasm for work,” “Work inspires me,” and “I am proud of my work.” Job fulfillment, job autonomy, and job consistency were assessed using subscales from the Nurse's Career Identity Scale (Chinese version), which was translated from the Japanese version of that scale. Job fulfillment was measured using the following three items (α = .755): “The work I do affects the state of the care recipients,” “I exert great influences on care recipients,” and “My job makes my care recipients get better.” Job autonomy was assessed using the following three items (α = .851): “I can decide how to work,” “I can arrange my own job,” and “I can work in my own way.” Job consistency was measured using the following three items (α = .867): “Nursing work suits me,” “Nursing is a part of my job,” and “I can take advantage of my strengths in my work.” All of the items were rated using a 7-point scale, ranging from *strongly disagree* (0) to *strongly agree* (6). The Cronbach's alpha for internal consistency and reliability was .910.

#### Job burnout

Job burnout was assessed using Maslach's Burnout Inventory-Human Service Survey (Maslach et al., 1986), which asks about the frequency of experiencing feelings related to the three dimensions of the burnout syndrome: emotional exhaustion, depersonalization, and reduced personal accomplishment. Emotional exhaustion was assessed using five items (α = .895), depersonalization was assessed using four items (α = .838), and reduced personal accomplishment was assessed using six items (α = .921). All of the items were scored on a 7-point frequency scale, ranging from 0 (*never*) to 6 (*every day*). The Cronbach's alpha for internal consistency and reliability was .887.

#### Pay satisfaction

Pay satisfaction was distinguished into the four dimensions of wage, insurance, welfare, and allowance satisfaction because employee pay in China generally consists of wage, insurance, welfare, and allowance. Pay satisfaction was assessed by asking the following items: “How satisfied are you with your current wages?”, “How satisfied are you with your insurance?”, “How satisfied are you with your subsidy status?”, and “How satisfied are you with your welfare?” These items were rated on a 5-point scale, ranging from *strongly dissatisfied* (1) to *strongly satisfied* (5). The Cronbach's alpha for internal consistency and reliability was .748.

### Data Analysis

Data analysis was performed using SPSS for Windows Version 20.0 (IBM, Inc., Armonk, NY, USA) and AMOS Version 21.0 (IBM, Inc., Armonk, NY, USA). The verification of the theoretical model was conducted in the two steps of measurement model analysis and structural model analysis. First, a confirmatory factor analysis was performed to test the measurement model. Factor loading, *R*^2^, and construct reliability were used to evaluate reliability, and the average variance extracted was used as an indicator of validity. All of the measurement models and factor structure models (intrinsic motivations) should be supported by investigation data.

Next, all of the testing models were qualified, and further structural model analysis was carried out. The structural equation model (SEM) between variables was analyzed to verify the hypotheses. The goodness-of-fit of the model was evaluated using the chi-square test statistic, adjusted goodness-of-fit index (AGFI), standardized root mean square residual (SRMR), root mean square error of approximation (RMSEA), comparative fit index (CFI), and Tucker–Lewis index (TLI). Values that were higher than .90 for AGFI, CFI, and TLI or lower than .08 for RMSEA and SRMR indicated acceptable model fit (Hair, 2011).

## Results

### Descriptive Analysis

Descriptive analyses for all variables are presented in Table 1. Most (84.4%) of the participants were female, the average age was about 49 years, 74.6% were from rural areas, and 84.9% lived with a spouse. With respect to education, 37% of the participants were illiterate, 37.8% were educated to the junior middle school level or lower, and only 25.2% had a senior high school or higher education. When asked about their health status, 92.6% of the participants reported being “relatively healthy” or “very healthy.” Most (84.0%) had no technical title, 50.7% were not certified, 41.5% had a nursing professional certificate, and 7.5% had a nurse's professional certificate. The average per capita of annual income of the participants was 34,200 RMB. Nearly all (97.9%) were full-time caregivers, they worked an average of 87.97 hours per week, and their average length of caregiving work experience was 61.29 months.

**Table 1:** Descriptive Statistics (N = 1,212)

Variable	*n*	%
Gender (female)	1,023	84.4
Age (17–72 years; *M* and *SD*)	49.2	10.4
Residence (rural)	904	74.6
Marital status (married)	1,029	84.9
Education		
Illiterate	448	37.0
Junior middle school or below	458	37.8
Senior middle school or above	306	25.2
Health status (healthy)	1,122	92.6
Technical title (no title)	1,018	84.0
Certificate		
No certificate	614	50.7
Nurse's professional certificate	91	7.5
Nursing professional certificate	503	41.5
Full-time	1,186	97.9
Variable (range)	*M*	*SD*
Annual income (RMB, 0–180,000)	34,200	16,900
Length care work career (months; 1–365)	61.29	62.60
Weekly working hours (8–168)	87.97	40.46
Pay satisfaction (1–5)		
Wage	3.36	0.93
Insurance	3.28	0.90
Welfare	3.42	0.91
Allowance	3.38	0.84
Job burnout (0–6)		
Emotional exhaustion	1.32	1.12
Depersonalization	0.76	0.94
Reduced personal accomplishment	1.50	1.30
Intrinsic motivation (0–18)		
Job dedication	12.85	3.37
Job fulfillment	13.65	2.91
Job autonomy	13.42	3.21
Job consistency	14.20	2.82

### Validity and Reliability

Convergent validity was tested using the guidelines recommended by Hair (2011). As shown in Table 2, all of the factor coefficients were strongly significant (*p* < .001). With the exception of the “reduced personal accomplishment” item, all item values were above the .50 cutoff value, as suggested by Hair et al. (2010), showing acceptable loadings. Despite its low factor loading, the “reduced personal accomplishment” item was retained as it was still significant (*p* < .001). The latent variable of job burnout explained 18.5% of the variance in the measurement items.

**Table 2:** Measurement Model: Standardized Factor Loadings, Validity, and Reliability

Construct Item	Standardized Factor Loading	*p*	*R*^2^	AVE	CR
Job dedication				.64	.84
I am full of enthusiasm for work.	.89	—	.79		
Work inspires me.	.71	< .001	.50		
I am proud of my work.	.78	< .001	.61		
Job fulfillment				.51	.75
The work I do affects the state of the care recipients.	.78	—	.61		
I exert great influences on care recipients.	.64	< .001	.51		
My job makes my care recipients get better.	.71	< .001	.41		
Job autonomy				.66	.85
I can decide how to work.	.80	—	.63		
I can arrange my own job.	.85	< .001	.73		
I can work in my own way.	.78	< .001	.61		
Job consistency				.69	.87
Nursing work suits me.	.83	—	.69		
Nursing is a part of my job.	.87	< .001	.76		
I can take advantage of my strengths in my work.	.78	< .001	.61		
Pay satisfaction				.45	.76
How satisfied are you with your current wages?	.57	< .001	.32		
How satisfied are you with your insurance?	.50	< .001	.25		
How satisfied are you with your subsidy status?	.75	< .001	.56		
How satisfied are you with your welfare?	.82	—	.68		
Job burnout				.44	.69
Emotional exhaustion	.68	< .001	.46		
Depersonalization	.82	—	.67		
Reduced personal accomplishment	.43	< .001	.19		
Intrinsic motivation				.75	.92
Job dedication	.66	< .001	.44		
Job fulfillment	.98	—	.97		
Job autonomy	.84	< .001	.71		
Job consistency	.94	< .001	.87		

***Note.*** AVE = average variance extracted; CR = construct reliability.

All of the average variance extracted values with the exception of job burnout (.44) and pay satisfaction (.45) scored above .5. The construct reliability value for each latent variable with the exception of job burnout (.69) scored above the cutoff value of .7. Therefore, as all of the values either met or approached the standards of experience value, all of the latent variables of the measurement models were determined as having strong reliability and convergent validity. Furthermore, as shown in Table 3, reasonable discriminant validity was obtained for intrinsic motivation, job burnout, and pay satisfaction.

**Table 3:** Discriminate Validity Among the Four Variables

Item	AVE	Intrinsic Motivation	Pay Satisfaction	Job Burnout
Intrinsic motivation	.74	.864		
Pay satisfaction	.45	.193***	.673	
Job burnout	.44	−.481***	−.201***	.663

***Note.*** Correlation matrix for the latent variables with square value of AVE on the diagonal. AVE = average variance extracted.

***Significance at the .001 level.

The confirmatory factor analysis models also obtained good results for model fit. For the factor analysis model of intrinsic motivation: CMIN/*df* = 5.96, CFI = .97, RMSEA = .06, TLI = .96, NFI = .96, GFI = .96, and SRMR = .04. For the measurement model of pay satisfaction: CMIN/*df* = 0.44, CFI = 1, RMSEA = .000, TLI = 1, NFI = .99, GFI = 1, and SRMR = .005. With respect to the latent variables of job burnout constructed by three factors, the measurement model was a saturated model, that is, just identified. Thus, good structural validity was also obtained for the measurement models of the three latent variables.

### Structural Model Analysis

The research hypotheses were evaluated using an SEM to test the theoretical model against the questionnaire results. One of the primary assumptions necessary for an SEM is that observed variables follow a multivariate normal distribution. If the data do not follow a multivariate normal distribution, the standard errors and hypothesis tests may be unreliable, which may be remedied using a bootstrap approach because this approach does not require assumptions regarding data distribution (Byrne, 2009). Because the data in this study did not follow a multivariate normal distribution (kurtosis = 154.48), a bootstrap approach was used to test the hypotheses within our SEM. The chi-square value obtained from the SEM analysis was strongly significant (χ^2^ = 927.81, *df* = 145, χ^2^/*df* = 6.40, *p* < .001). In addition, other fit indices (AGFI = .90, SRMR = .08, RMSEA = .07, CFI = .93, TLI = .92) were examined, with results showing that the structural model had a good fit.

The results of the structural path model are shown in Table 4. Intrinsic motivation was shown to relate positively to pay satisfaction (β = .11, *p* < .05) and to relate negatively to job burnout (β = −.46, *p* < .01). Job burnout was shown to relate negatively to pay satisfaction (β = −.13, *p* < .01). Therefore, all of the hypotheses were supported, and job burnout was shown to play a significant, mediating role in the relationship between intrinsic motivation and pay satisfaction (β = .06, *p* < .01).

**Table 4:** Parameter Estimates of the Hypothesized Model of Influencing and Mediating Factors of Pay Satisfaction

Path	Standardized Factor Loading	Bias-Bootstrap 2000		Factor Loading	Bias-Bootstrap 2000		Hypothesis
		95% CI	*p*		95% CI	*p*	
Intrinsic motivation on pay satisfaction	.13	[.026, .209]	< .05	.11	[.024, .188]	< .05	Supported
Intrinsic motivation on job burnout	−.48	[−.617, −.384]	< .01	−.46	[−.563, −.366]	< .01	Supported
Job burnout on pay satisfaction	−.14	[−.242, −.056]	< .01	−.13	[−.240, −.048]	< .01	Supported
Direct effect				.11	[.024, .188]	< .05	Supported
Indirect effect				.06	[.022, .124]	< .01	Supported
Total effect				.17	[.107, .237]	< .01	Supported
Indirect effect/total effect				.35			

## Discussion

To the authors' knowledge, this is the first study to investigate the relationship between intrinsic motivation and pay satisfaction among caregivers employed in RHE in China. Four interesting findings in this study are presented below.

The results confirm the first hypothesis that intrinsic motivation positively relates to pay satisfaction among caregivers employed in RHE, indicating that caregivers with higher levels of intrinsic motivation are more satisfied with their pay and are more likely to accept lower pay. This result is consistent with the theoretical framework of compensating differentials and with previous studies that found that low pay for care work may be compensated in part by intrinsic rewards (Macdonald & Merrill, 2002). The job of elderly care in residential homes requires that caregivers care for fictive parents within the context of traditional Chinese filial-piety values (Yun-Xiu et al., 2013). Thus, the overlapping roles of paid caregiver and “child” (in the fictive child–parent relationship) establish a kin-like relationship between caregivers and care recipients (Dodson & Zincavage, 2007). Therefore, caregivers are more likely to engage proactively in caregiving work and to gain a positive emotional experience that compensates somewhat for low pay. The effect of intrinsic motivations dampens the effect of external motivations such as compensation in caregivers working in RHE. However, the qualitative findings of this study indicate that the effect of intrinsic motivation is an excuse that caregivers use to justify their wage penalty and acclimate to their role as a “prisoner of love” (Folbre, 2001) rather than bargaining for higher pay. Therefore, if caregivers are motivated only by intrinsic rewards, they are more likely to allow employers to pay them less.

The results also support the second hypothesis that intrinsic motivation negatively relates to job burnout in caregivers employed in RHE, echoing the findings of previous studies (Kuvaas et al., 2017). This implies that caregivers who are mainly intrinsically motivated may experience low levels of job burnout. The major symptoms of job burnout are loss of energy and mastery, lack of motivation, and negative attitudes toward others (Maslach et al., 1986). Intrinsically motivated caregivers are more likely to have the right to choose and to make decisions, devote their hearts and hands, and experience positive emotions. Therefore, higher levels of intrinsic motivation confer better psychological and physical well-being and a better capacity to deal with stress, which helps ameliorate job burnout even under conditions of low pay (Gagné et al., 1997).

The results also support the third hypothesis that job burnout relates negatively to pay satisfaction in caregivers employed in RHE, with higher levels of job burnout associated with lower levels of pay satisfaction. A possible reason for this may be that caregivers with high levels of job burnout perceive their job as a source of disamenity and thus expect a pay premium based on the theoretical framework of compensating differentials. Caregivers generally work long hours per week and care for many residents in poor working conditions while being compensated poorly, all of which may negatively affect their pay satisfaction.

The study further revealed that job burnout plays a significant, mediating role in the relationship between intrinsic motivation and pay satisfaction among caregivers employed in RHE, which supports Hypothesis 4. In other words, caregivers with higher levels of intrinsic motivation and lower levels of job burnout have higher levels of pay satisfaction. Thus, promoting intrinsic motivation while reducing job burnout may enhance pay satisfaction.

This research has profound implications for nursing education, practice, and research. First, more attention should be paid to instill nursing values, altruism, identification, and commitment in caregivers to foster their intrinsic motivation to prepare them to better handle job burnout in their professional practice. Second, nonpecuniary rewards such as occupational prestige and personal honor may be offered to caregivers by the government and managers to acknowledge the value of their care work and strengthen their intrinsic motivation. In addition, traditional filial piety culture should be used by managers to encourage caregivers to establish kin-like relationships with their care recipients, which may further stimulate their intrinsic motivation. Third, a supportive working climate, including high levels of job autonomy, more advancement opportunities, reasonable workloads, and role stress moderation, should be fostered by policymakers and organization managers to reduce job burnout. Furthermore, family-friendly organizational policies, including flexible work scheduling, child/elder care assistance, more vacation, flexible work arrangements, and more subsidies (Brough et al., 2005), should be implemented to help caregivers balance work and family, which may further enhance intrinsic motivation. Fourth, caregivers employed in RHE in China are paid significantly less than workers in government agencies. Although no national data are available, data from Jiangsu Province (a developed province in eastern China; 84,688 yuan per year; Jiangsu Province Bureau of Statistics, 2017), Hubei Province (a moderately developed province in central China; 78,423 yuan per year; Hubei Province Bureau of Statistics, 2017), and Guizhou Province (a less-developed province in western China; 71,795 yuan per year; Guizhou Province Bureau of Statistics, PRC, 2017) indicate that all compensate government workers at a rate that is over twice as high as the average earned by the caregivers in this sample. Therefore, as the positive influence of intrinsic motivations on pay satisfaction may obscure the wage penalty currently experienced by caregivers, it is necessary to increase the awareness of caregivers regarding their rights to decent pay and to promote their ability to bargain collectively through strong unions. Fifth, appropriate public policies should be implemented to recognize and appreciate the intrinsic motivation of caregivers to promote their pay satisfaction. Several suggestions include providing wage subsidies to caregivers to increase their pay, implementing government purchase and long-term care insurance programs to improve the ability of low-income residents to pay for caregiving services, and forming and empowering unions to promote the collective bargaining ability of caregivers employed in RHE. Sixth, pay should be provided as an acknowledgment and appreciation for caregiving services rather than as a locus of control. Therefore, as a typical representative of extrinsic motivation, pay could be internalized and crowds in intrinsic motivation of caregivers (Nelson & Folbre, 2006).

### Conclusions

This study investigated the influences of intrinsic motivation on pay satisfaction among caregivers employed in RHE in China. The findings indicate that intrinsic motivation relates positively to pay satisfaction, which is consistent with the theoretical framework of compensating differentials, under which nonpecuniary benefits compensate somewhat for low pay. Moreover, the findings of this study confirmed that job burnout plays a mediating role in the relationship between intrinsic motivation and pay satisfaction.

### Limitation and Future Studies

The study was methodologically limited by the cross-sectional design used to assess structural models, including the mediating model (Cole & Maxwell, 2003). In future studies, researchers should employ a longitudinal study design to facilitate causal inferences. In addition, this study examined only the role of job burnout as a mediator between intrinsic motivation and pay satisfaction. The many other possible mediating variables were not considered. Therefore, more mediating variables should be examined to obtain a more comprehensive understanding of this relationship. Moreover, the chi-square used in this study did not reach the standard of experience value. Although a chi-square test is commonly used for evaluating model fit in structural equation modeling (Kline, 2015), it is very sensitive to sample size. If the sample size is large, the chi-square statistic may be significant although differences between observed and model-implied covariances are slight (Chen et al., 2016). Thus, the results of this study should be interpreted with caution. In addition, the small number of male caregivers in our sample obviated the potential for conducting a comparative study on gender-related differences. Samples that include a sufficient number of male caregivers are necessary in future research. Finally, this study only examined the influences of intrinsic motivation on pay satisfaction. Future studies should further investigate the influences of pay satisfaction on intrinsic motivation based on the motivation crowding theory.
